# Effects of exercise interventions on adolescents with mild to moderate scoliosis: a meta-analysis

**DOI:** 10.7717/peerj.21355

**Published:** 2026-08-06

**Authors:** Qing Liu, Chengli Xu, Jia Li, Jing Tian, Chenxu Xue, Tenghao Yu, Kuan Dong

**Affiliations:** 1 School of Physical Education, Central China Normal University, Wuhan, China; 2 Hebei Academy of Fine Arts, Shijiazhuang, China; 3 College of Life Science, Henan Normal University, Xinxiang, China

**Keywords:** Exercise interventions, Adolescent scoliosis, Cobb angle, Axial trunk rotation, Quality of life, Meta-analysis

## Abstract

**Background:**

Adolescent idiopathic scoliosis (AIS) is a prevalent spinal deformity that impairs physical function and quality of life. While supervised exercise is recommended for moderate cases, its effectiveness remains debated. This meta-analysis evaluates the impact of exercise therapy on Cobb angle, trunk rotation (ATR), and quality of life (QoL) in AIS patients.

**Methods:**

We systematically searched PubMed, Web of Science, EBSCO and China National Knowledge Infrastructure (CNKI) databases from inception to October 2025 for randomized controlled trials (RCTs) investigating exercise in AIS. Study quality was assessed using the PEDro scale. Data synthesis involved pooling effect sizes, conducting subgroup analyses, and assessing publication bias using Stata 18.

**Results:**

A total of 26 studies (21 RCT and five controlled trials) were included in the meta-analysis. The exercise intervention demonstrated statistically significant improvements across multiple functional domains: reduced the Cobb angle (*Hedge’s* = 1.22, 95% CI [0.90–1.53]), improved the ATR (*Hedge’s* systematically searched 1.25, 95% CI [0.74–1.76]), and enhanced QoL (*Hedge’s* systematically searched 0.73, 95% CI [0.38–1.09]). Subgroup analyses further indicated that Schroth-based training (*Hedge’s* = 1.304) and combined approaches (*e.g.*, exercise with orthotic management) yielded the most favorable outcomes. Importantly, both short-term (≤12 weeks, 1–2 sessions/week) and long-term (≥25 weeks, ≥5 sessions/week) intervention durations demonstrated significant clinical efficacy.

**Conclusion:**

Exercise therapy demonstrates significant potential as a fundamental conservative intervention for improving spinal morphology, function, and quality of life in adolescents with idiopathic scoliosis. The current evidence supports its integration into primary clinical management protocols. Future research should focus on conducting multi-center randomized controlled trials to validate long-term efficacy and to establish optimal, individualized intervention protocols.

## Introduction

Adolescent idiopathic scoliosis (AIS) is a common three-dimensional spinal deformity characterized by lateral curvature and vertebral rotation, accounting for approximately 90% of diagnosed scoliosis cases in adolescents (Qiu, 2017). With a reported prevalence ranging from 0.47% to 5.2%, AIS represents one of the most prevalent growth-related musculoskeletal conditions in this population (Weiss et al., 2006). The deformity typically emerges during the prepubertal period and may progress rapidly throughout pubertal growth. Although incidence rates are similar between sexes, curve progression tends to be more pronounced in females. The etiology of scoliosis is multifactorial, including congenital, idiopathic, neuromuscular, and functional subtypes. If not adequately managed, AIS can lead to persistent structural and functional impairments that extend into adulthood, while also exerting considerable physiological and psychological impacts on affected individuals (Kuznia, Hernandez & Lee, 2020; Negrini et al., 2018).

According to the International Society on Scoliosis Orthopaedic and Rehabilitation Treatment (SOSORT), management of AIS should be tailored to curvature severity: observation or scoliosis-specific exercises are recommended for Cobb angles <25°; bracing combined with specific exercises is advised for curves between 25° and 45°; and surgical intervention is generally indicated for Cobb angles exceeding 45° (Negrini et al., 2015). In Europe, several evidence-based physiotherapeutic approaches have been developed, including the Schroth method (Germany), Scientific Exercises Approach to Scoliosis (SEAS), Barcelona Scoliosis Physical Therapy School (BSPTS), Functional Individual Therapy of Scoliosis (FITS), as well as FED, Lyon, Side Shift, and Dob Med therapies (Seleviciene et al., 2022). Global postural reeducation (GPR), a manual therapy approach focusing on stretching muscle chains and postural restoration, has demonstrated potential benefits in reducing Cobb angle progression and improving postural alignment in adolescents with idiopathic scoliosis when compared to standard care (Hospital, 2024).

Clinical studies report that 27% to 59% of patients with AIS experience pain, alongside reductions in muscle strength, functional capacity, and respiratory muscle performance (Bettany-Saltikov et al., 2015; Ceballos-Laita et al., 2023; Théroux et al., 2017; Weinstein, 2019). These functional impairments highlight the importance of integrating targeted exercise therapy into comprehensive management protocols.

AIS is characterized by a three-dimensional spinal deformity involving vertebral rotation, thoracic structural changes, postural asymmetry, proprioceptive deficits, and impaired motor control. These structural alterations can compromise cardiopulmonary development and function—particularly in severe cases—by affecting ventilation and gas exchange (Hong et al., 2011). The Cobb angle serves as the gold standard for quantifying the magnitude of spinal curvature in AIS, though responsiveness to non-surgical interventions may vary considerably. Given the substantial impact of AIS on adolescent health and development, patient-reported quality of life (QoL) represents a critical outcome domain, most commonly assessed using the validated Scoliosis Research Society-22 (SRS-22) questionnaire (Asher et al., 2003).

Current evidence suggests that the Schroth method demonstrates greater efficacy in reducing Cobb angle, improving trunk rotation, and enhancing QoL compared to no intervention or other conservative treatments (Ceballos Laita et al., 2018). Recent studies further indicate that while combined approaches integrating Schroth-based exercises with complementary techniques significantly improve postural alignment and trunk asymmetry, the standalone Schroth method appears particularly effective in enhancing quality of life outcomes (Dong et al., 2024). A recent network meta-analysis has provided valuable insights into the comparative effectiveness of different exercise modalities for AIS, demonstrating that Schroth therapy may be most effective for reducing Cobb angle, combined interventions for improving trunk rotation (ATR), and core stability training for enhancing QoL (Zhu et al., 2025). However, as a network meta-analysis, their work focuses on ranking interventions based on relative efficacy but does not systematically examine how intervention characteristics—such as frequency, duration, and total sessions—moderate these treatment effects. This gap limits the ability of clinicians to translate comparative efficacy rankings into specific, actionable prescription guidelines.

Therefore, this systematic review and meta-analysis aims to comprehensively evaluate the effects of exercise-based interventions on Cobb angle, ATR, and QoL in adolescents with mild to moderate idiopathic scoliosis. Furthermore, it seeks to explore the moderating effects of intervention characteristics, including type, duration, and frequency to identify optimal therapeutic strategies.

## Methods

This systematic review was conducted in accordance with the Preferred Reporting Items for Systematic Reviews and Meta-Analyses (PRISMA) 2020 statement (Table S1). The review protocol was prospectively registered with the International Prospective Register of Systematic Reviews (PROSPERO) under registration number CRD420251030156 (Table S2).

### Search strategy

Searches were conducted in PubMed, Web of Science, EBSCO and China National Knowledge Infrastructure. The search included studies published up to February 2025, and all search results were imported into EndNote 20. This meta-analysis synthesized data from previously published studies and did not involve original experimental research.

Two researchers (QL and JL) independently conducted the literature search and screening process. Duplicate records were first removed using EndNote’s built-in function. Next, titles and abstracts were screened for relevance. Articles were then assessed against the study’s inclusion and exclusion criteria. Finally, the full texts of the remaining articles were reviewed to determine eligibility for inclusion.

The search algorithm included all possible keyword: (“Adolescent” OR “teen” OR “boy” OR “girl” OR “teenager” OR “child” OR “young adult”) AND (“Scoliosis” OR “idiopathic scoliosis” OR “adolescent idiopathic scoliosis” OR “AIS”) AND (“Exercise” OR “train” OR “sport” OR “physical activity” OR “motion” OR “locomotion” OR “athletic” OR “gymnastic” OR “motor” OR “physiotherapeutic scoliosis specific exercise” OR “PSSE” OR “Schroth” OR “Barcelona Scoliosis Physical Therapy School” OR “BSPTS” OR “Lyon” OR “scientific exercises approach scoliosis” OR “SEAS” OR “DoboMed” OR “Side-shift” OR “functional individual therapy scoliosis” OR “FITS” OR “physiotherapy”) (Table S3).

### Inclusion criteria

Studies were included based on the following PICOS criteria: (1) adolescents (typically defined as aged 10–18 years) with a diagnosis of mild to moderate AIS, confirmed by a Cobb angle between 10 and <45. (2) The intervention involved structured, supervised exercise-based interventions, including but not limited to core stabilization training, functional orthopedic exercises, Schroth therapy, proprioceptive training, or other physiotherapeutic scoliosis-specific exercise (PSSE) programs. (3) The control group received alternative conservative treatments (*e.g.*, bracing, passive manual therapy, general stretching, observation/usual care) or no intervention. (4) The primary outcome was change in the Cobb angle (measured in degrees), secondary outcomes included changes in the angle of ATR and disease-specific QoL, assessed using validated instruments such as the Scoliosis Research Society-22 (SRS-22) questionnaire. (5) The study design was controlled trials, including both randomized control trials (RCTs) and non-randomized designs, were eligible.

### Exclusion criteria

Studies were excluded according to the following criteria: (1) duplicate publications or overlapping datasets; (2) publications in languages other than English or Chinese; (3) unavailability of the full text; (4) incomplete, inconsistent, or non-extractable outcome data; (5) studies investigating exercise interventions delivered exclusively during the postoperative period; (6) animal studies, pharmacological trials, or retrospective clinical designs. In cases where studies met all inclusion criteria but did not present extractable outcome data in the full text, they were recorded in the PRISMA flow chart and discussed qualitatively. Such studies were not included in the quantitative synthesis, and a narrative assessment of their potential impact on the body of evidence is provided in the discussion. In addition, studies where the primary research question focused on comparing exercise to non-exercise interventions (*e.g.*, bracing alone) rather than comparing different exercise modalities were considered for exclusion, as they address a different clinical question and introduce significant heterogeneity into the pooled analysis of exercise type comparisons.

### Methodological quality assessment

The PEDro scale was used to evaluate methodological quality of the included studies. It consists of 11 criteria addressing key domains of trial design and reporting, including random allocation, blinding procedures, and baseline comparability. Studies are scored on a scale from 0 to 10, with higher scores indicating better methodological quality. Based on established cut-off values (Lee et al., 2024), studies are categorized as low quality (≤4 points), moderate quality (5–6 points), or high quality (≥7 points). This process was carried out by two independent reviewers (QL, XCX). Conflicts were resolved by discussion within the review team.

### Statistical analysis

Stata 18 was used to perform effect size pooling, forest plot generation, and subgroup analyses. Continuous outcome data were extracted as means and standard deviations. Due to anticipated clinical and methodological variability across studies, a random-effects model was applied a priori for all meta-analyses to account for between-study heterogeneity (O’Connor, Green & Higgins, 2008). Heterogeneity was quantified using the I^2^ statistic, interpreted as follows: 25–49% low, 50–74% moderate, and ≥75% high (Higgins et al., 2011).

Effect sizes were computed as Hedge’s with 95% confidence intervals (CIs) (Hermes & Fry, 2023), incorporating a correction for small-sample bias. Based on conventional thresholds, effect sizes were categorized as very small (≤0.19), small (0.20–0.59), medium (0.60–1.19), or large (≥1.20) (Hopkins et al., 2009). Publication bias was assessed through visual inspection of funnel plots (Sedgwick & Marston, 2015) and, where more studies were available for an outcome, supplemented by Egger’s regression test (Egger et al., 1997).

Meta-regression was performed to examine the influence of predefined moderator variables on effect estimates, thereby exploring potential sources of heterogeneity (Ravishankar & Sreekumaran Nair, 2016). Moderators were selected based on theoretical relevance and prior evidence indicating their possible role in influencing treatment outcomes (Cui, Chen & Su, 2018). Sensitivity analyses were conducted using the leave-one-out method to evaluate whether the overall results were disproportionately affected by any single study.

## Results

### Search results

**Figure 1:**
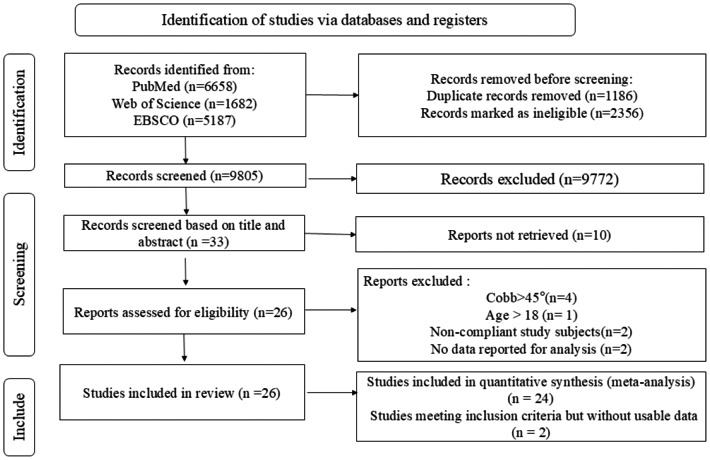
Flow chart for inclusion and exclusion of studies.

A total of 13,529 articles were retrieved from five databases. After duplicates were removed (*n* = 3,372) using EndNote 20, 9,815 records remained. A preliminary screening of titles and abstracts excluded 9780 records, leaving 35 articles for full-text assessment. During the full-text evaluation for eligibility, nine studies were excluded based on predefined eligibility criteria. Ultimately, 26 clinical trials published between 2014 and 2025 were included in this meta-analysis, comprising 21 randomized controlled trials (RCTs) and five clinical controlled trials (CCTs), as illustrated in Fig. 1 chart for inclusion and exclusion of studies. Of these, 24 studies reported outcome data suitable for quantitative synthesis and were included in the meta-analysis. The remaining two eligible RCTs (Abbott, Möller & Gerdhem, 2013; Charalampidis et al., 2024) met the inclusion criteria but did not report extractable outcome data (Charalampidis et al., 2024) compared nighttime bracing, physical activity, and scoliosis-specific exercises in patients with moderate AIS, while (Abbott, Möller & Gerdhem, 2013) published a study protocol without completed outcome data. Despite attempts to contact the authors, raw data could not be obtained; therefore, these two studies are described qualitatively but were excluded from the quantitative synthesis.

### Characteristics of included studies

This study included a total of 25 clinical trials (Abbott, Möller & Gerdhem, 2013; Charalampidis et al., 2024; Duangkaew et al., 2020; Gao et al., 2019; Gür, Ayhan & Yakut, 2017; Ji, 2020; Khaledi et al., 2024; Kim & HwangBo, 2016; Kim & Park, 2017; Kocaman et al., 2021; Küçük, Öten & Coşkun, 2024; Kwan et al., 2017; Lau et al., 2021; Mohamed & Yousef, 2021; Monticone et al., 2014; Park et al., 2021; Radwan, Ibrahim & Mahmoud, 2022; Schreiber et al., 2016; Schreiber et al., 2015; Wang, Lu & Wang, 2024; Yagci, Ayhan & Yakut, 2018; Yagci & Yakut, 2019; Zapata et al., 2023; Zhang et al., 2024; Zheng et al., 2018). The sample sizes ranged from six to 80 cases. The majority of the participants in this study were adolescents with AIS, ranging in age from 10 to 18 years. Intervention strategies encompassed multiple corrective exercise approaches. Specifically, 10 studies employed Schroth three-dimensional corrective training, often incorporating adjunctive techniques such as bracing or proprioceptive training. Five studies focused on core stability training, which aimed to activate deep trunk muscles, including the transverse abdominis and multifidus. Additionally, six studies evaluated the efficacy of orthoses alone (*e.g.*, TLSO braces) in comparison to combined orthosis-exercise interventions. Training duration varied substantially across the included studies, ranging from 4 weeks to 12 months. Frequency is typically 2 to 5 times per week, with each session lasting 40 to 90 min. Some treatment programs include outpatient guidance and home exercises (*e.g.*, 40 min of clinical therapy per week, plus 10 to 15 min of daily home practice).

Outcome measures focused on health impacts (13 items related to QoL, functional assessment (13 items evaluating sacroiliac joint mobility), and structural improvement (22 items using Cobb angle measurements). Compared to control groups primarily receiving standard rehabilitation therapy, orthotic devices alone, or observational follow-up, five studies specifically examined innovative combined therapies, such as treatment regimens integrating Schroth therapy with proprioceptive neuromuscular facilitation (PNF) or spinal mobilization techniques. Current research primarily focuses on the synergistic effects of orthoses and exercise therapy; however, long-term follow-up (>1 year) and standardized dose effects require further validation (Table 1).

**Table 1:** Study characteristics and intervention details.

Author (year)	Design	Groups (n)	Age (Years)	Diagnosis (Cobb angle)	Intervention duration & frequency	Intervention content	Outcomes
Küçük, Öten & Coşkun (2024)	RCT	20 *vs* 20	10–16	10°–25°	10 weeks, 2 ×/week	Mobilization + CSE *vs* CSE	Cobb, ATR
Gao et al. (2019)	RCT	23 *vs* 22	>10	25°–40°	6 months: Clinic every 2–3 months + daily home exercises	Orthosis + SSE *vs* TLSO	Cobb
Kim & HwangBo (2016)	CCT	12 *vs* 12	13–17	18°–30°	12 weeks, 3 ×/week, 1 h/session	Schroth *vs* Pilates	Cobb
Gür, Ayhan & Yakut (2017)	RCT	12 *vs* 13	10–16	>20°	10 weeks, 2 ×/week, 1 h/session	Core stabilization *vs* traditional rehab	Cobb, ATR, QoL
Yagci, Ayhan & Yakut (2018)	CCT	10 *vs* 10	10–16	NR	10 weeks, 60 min/session	BBAT *vs* TE	Cobb, ATR, QoL
Yagci & Yakut (2019)	RCT	15 *vs* 15	≥12	30°–45°	4 months, daily sessions	Brace + Core *vs* Brace + SEAS	Cobb, ATR, QoL
Kocaman et al., (2021)	RCT	14 *vs* 14	10–18	10°–26°	10 weeks, 3 ×/week, 90 min/session	Schroth *vs* Core training	Cobb, ATR, QoL
Park et al. (2021)	RCT	14 *vs* 14	14–18	>10°	8 weeks, 3 ×/week	Gymball + DNS *vs* Gymball	Cobb
Ji (2020)	RCT	11 *vs* 13	7–13	Mild idiopathic scoliosis	12 weeks, 4 ×/week, 1 h/session	Progressive stabilization training *vs* Follow-up observation	Cobb, QoL
Zapata et al. (2023)	RCT	28 *vs* 18	10–16	Mild (12°–24°)	1 year, 5 ×/week, 15 min/day	Schroth + HEP *vs* Control	Cobb
Kwan et al. (2017)	CCT	25 *vs* 24	10–16	25°–40°	8 weeks	Schroth *vs* Brace	ATR, QoL
Monticone et al. (2014)	RCT	55 *vs* 55	10.2–14.7	Mild (10°–25°)	80 min/week clinic + home sessions	Task-oriented exercises *vs* traditional rehab	Cobb, ATR, QoL
Kim & Park (2017)	RCT	8 *vs* 7	–	10°	60 min/session (frequency NR)	SERME *vs* SE	Cobb
Radwan, Ibrahim & Mahmoud (2022)	CCT	20 *vs* 20	10–16	10°–30°	3 months, 3 ×/week	Schroth *vs* Control	Cobb
Mohamed & Yousef (2021)	RCT	17 *vs* 17	14–16	<25°	6 months, 3 ×/week, 1 h/session	Schroth *vs* PNF	Cobb, ATR
Duangkaew et al. (2020)	RCT	10 *vs* 6	10–18	ATR > 7°	6 weeks, 2 ×/week	KT + Schroth *vs* Schroth	ATR
Lau et al. (2021)	RCT	14 *vs* 16	11–14	15°–40°	6 months, 5 ×/week, 7 min/day	Home-based E-Fit *vs* Control	QoL
Schreiber et al. (2015)	RCT	25 *vs* 25	10–18	10°–45°	6 months: Initial intensive + weekly sessions	Schroth *vs* Observation/brace	QoL
Schreiber et al. (2016)	RCT	25 *vs* 25	10–18	10°–45°	6 months: Initial intensive + weekly sessions	Schroth *vs* Observation/brace	Cobb
Zhang et al. (2024)	RCT	21 *vs* 21	10–18	10°–25°	24 weeks	Schroth + PNF *vs* PNF	Cobb, ATR, QoL
Zheng et al. (2018)	RCT	24 *vs* 29	10–17	20°–40°	12 months: Bi-monthly clinic + home exercises	SEAS *vs* Brace	Cobb, QoL
Wang, Lu & Wang (2024)	RCT	40 *vs* 80	10–17	>10°	4 weeks, 5 ×/week	Schroth + Laser *vs* Schroth	Cobb, ATR
Khaledi et al. (2024)	RCT	15 *vs* 15 *vs* 10	12–18	10°–40°	12 weeks, 3 ×/week	SE *vs* SE+ASSE *vs* No intervention	Cobb ATR QoL
Charalampidis et al. (2024)	RCT	45 *vs* 45 *vs* 45	9–17	25°–40°	5 years	NB *vs* PA *vs* SSE	Cobb
Abbott, Möller & Gerdhem (2013)	RCT	45 *vs* 45 *vs* 45	9–17	25°–40°	6 months	AS-PA *vs* SPE *vs* HNB	Cobb QoL

**Notes.**

RCT Randomized Controlled Trial CCT Controlled Clinical Trial SSE Scoliosis-Specific Exercise TLSO thoracolumbosacral orthosis SEAS Scientific Exercise Approach to Scoliosis PNF Proprioceptive Neuromuscular Facilitation BBAT Basic Body Awareness Therapy TE Traditional Exercises HEP Home Exercise Program DNS Dynamic Neuromuscular Stabilization CSE Core Stabilization Exercises KT Kinesio Taping SE Schroth exercises ASSE Asymmetric Spinal Stabilization Exercise ATR Angle of Trunk Rotation QoL Quality of Life NR Not Reported NB nighttime bracing PA nighttime bracing SSE scoliosis-specific exercise AS-PA adequate self-mediated physical activity SPE scoliosis specific exercises HNB hyper-corrective night-time brace

It should be noted that while (Charalampidis et al., 2024) and (Abbott, Möller & Gerdhem, 2013) met all inclusion criteria, they could not be included in the meta-analysis due to the unavailability of extractable outcome data. Charalampidis et al. (2024) represents a high-quality RCT comparing nighttime bracing *versus* exercise interventions, and its findings are discussed narratively in relation to our pooled results. Abbott, Möller & Gerdhem (2013) provides the protocol for the CONTRAIS trial, which informs the methodological context for ongoing research in this area.

### Risk of bias of included studies

For the following PEDro items, all studies included in the syntheses were considered at low risk of bias. Scores ranged from 4 to 9 out of 10. Most studies met criteria related to randomization, baseline comparability, dropout rate, intention-to-treat analysis, between-group comparison, and reporting of variability. Common limitations included inadequate reporting or implementation of allocation concealment and blinding procedures (participant, therapist, and assessor). The methodological quality is shown by means of a percentage diagram in Table 2.

**Table 2:** Scores of PEDro scale of included literatures.

Author (Year)	1	2	3	4	5	6	7	8	9	10	11	Total
Küçük, Öten & Coşkun (2024)	✓	✓	✓	✓				✓	✓	✓	✓	8
Gao et al. (2019)	✓	✓	✓	✓	✓	✓		✓	✓	✓	✓	9
Kim & HwangBo (2016)	✓			✓					✓	✓		4
Gür, Ayhan & Yakut (2017)	✓			✓	✓	✓		✓	✓	✓	✓	8
Yagci, Ayhan & Yakut (2018)	✓		✓	✓				✓	✓	✓	✓	7
Yagci & Yakut (2019)	✓		✓	✓				✓	✓	✓	✓	7
Kocaman et al., (2021)	✓			✓				✓	✓	✓	✓	6
Park et al. (2021)	✓		✓	✓				✓	✓	✓	✓	7
Ji (2020)	✓			✓				✓		✓	✓	5
Zapata et al. (2023)	✓	✓	✓	✓	✓	✓		✓	✓	✓	✓	9
Kwan et al. (2017)	✓	✓	✓	✓				✓	✓	✓	✓	8
Monticone et al. (2014)	✓	✓		✓	✓	✓		✓	✓	✓	✓	9
Kim & Park (2017)	✓		✓	✓				✓	✓	✓	✓	7
Radwan, Ibrahim & Mahmoud (2022)	✓		✓	✓				✓	✓	✓	✓	7
Mohamed & Yousef (2021)	✓			✓				✓	✓	✓	✓	6
Duangkaew et al. (2020)	✓	✓	✓	✓				✓	✓	✓		7
Lau et al. (2021)	✓	✓		✓	✓	✓		✓	✓	✓	✓	9
Schreiber et al. (2015)	✓	✓	✓	✓				✓	✓	✓	✓	8
Schreiber et al. (2016)	✓	✓	✓	✓	✓	✓		✓	✓	✓	✓	9
Zhang et al. (2024)	✓	✓	✓	✓				✓	✓	✓	✓	8
Zheng et al. (2018)	✓	✓		✓	✓	✓		✓	✓	✓	✓	9
Wang, Lu & Wang (2024)	✓			✓					✓	✓	✓	5
Khaledi et al. (2024)	✓	✓		✓				✓	✓	✓	✓	7
Charalampidis et al. (2024)	✓	✓	✓	✓			✓	✓	✓	✓	✓	9
Abbott, Möller & Gerdhem (2013)	✓	✓	✓	✓			✓		✓	✓	✓	8

**Notes.**

1, Qualification Standards; 2, Random Allocation; 3, Assign hidden; 4, Baseline Consistency; 5, Subject blinding method; 6, Therapist’s Blind Method; 7, Evaluator blind method; 8, The dropout rate of subjects should be less than or equal to 15%; 9, Intent Analysis; 10, Between-group statistical comparison; 11, Measurement of Variability.

### Results of quantitative synthesis

#### Cobb

**Figure 2:**
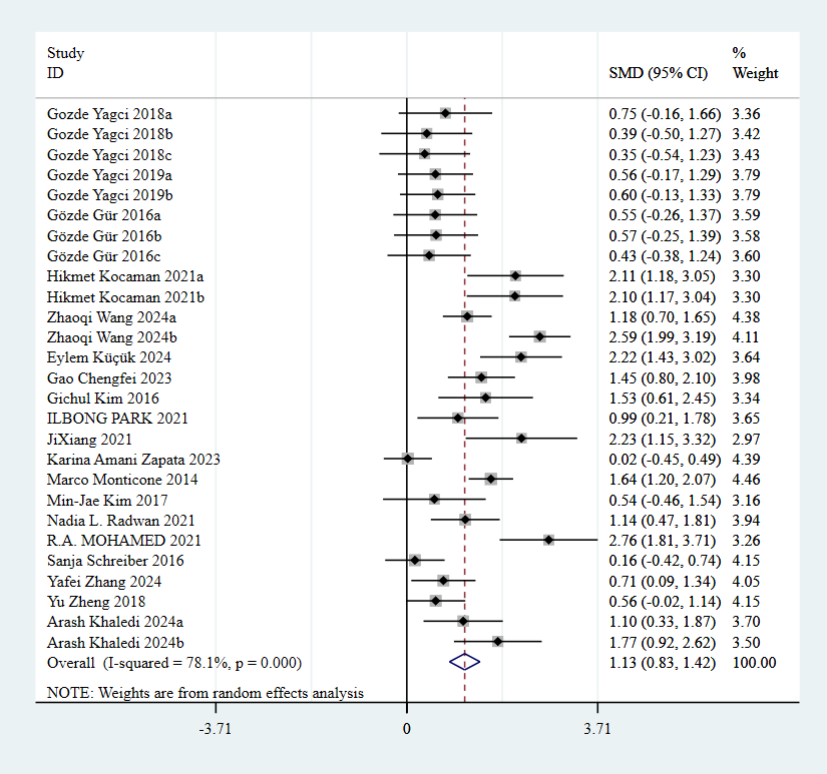
The forest plot of Cobb angle.

Twenty studies reported Cobb angle measurements across thoracic, lumbar, or total spinal regions, providing 26 analyzable data points for quantitative synthesis. Among these, five studies focused on thoracic curvature, five on lumbar curvature, and 16 reported overall spinal curvature. Exercise intervention demonstrated a statistically significant reduction in Cobb angle in adolescents with scoliosis, with a large pooled effect size (*Hedge’s* = 1.22, 95% CI [0.90–1.53]). Substantial heterogeneity was observed across studies (I^2^ = 1.2% in Fig. 2.

#### ATR

**Figure 3:**
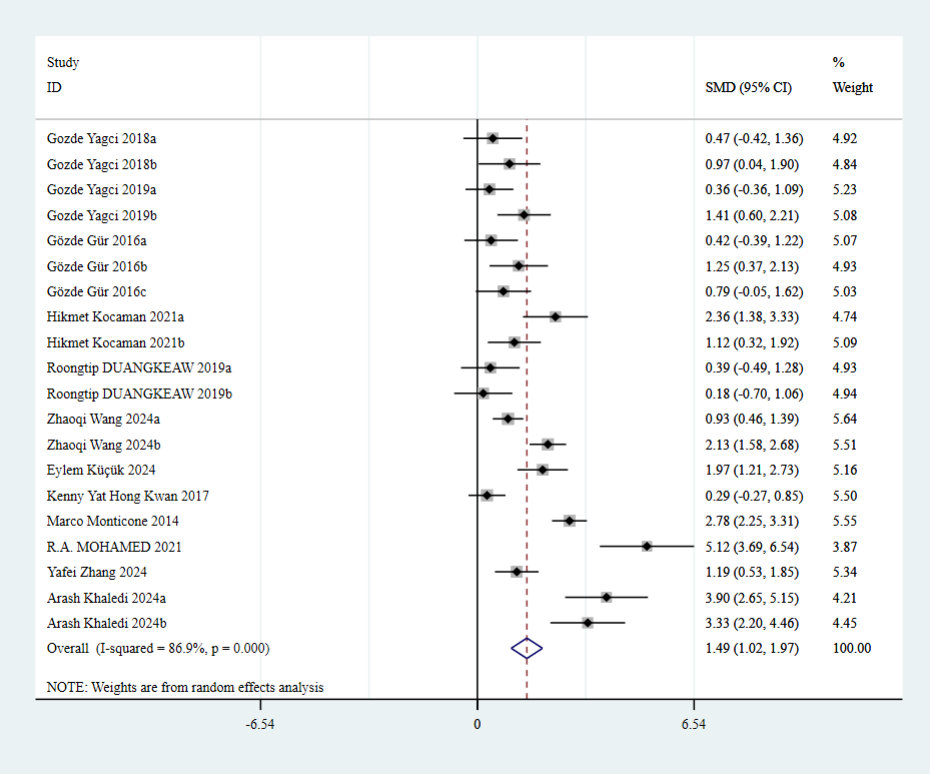
The forest plot of ATR.

Analyses of trunk rotation outcomes included data from six studies reporting thoracic rotation, six reporting lumbar rotation, and eight reporting overall trunk rotation. Exercise interventions demonstrated a significant overall reduction in spinal trunk rotation angle, with a large pooled effect size (*Hedge’s* = 1.25, 95% CI [0.74–1.76]). Considerable heterogeneity was observed across these studies (I^2^ = 89%). Despite this variability, exercise therapy showed a consistent and statistically significant improvement in trunk rotation between pre- and post-intervention assessments in adolescents with scoliosis (Fig. 3).

#### QoL

**Figure 4:**
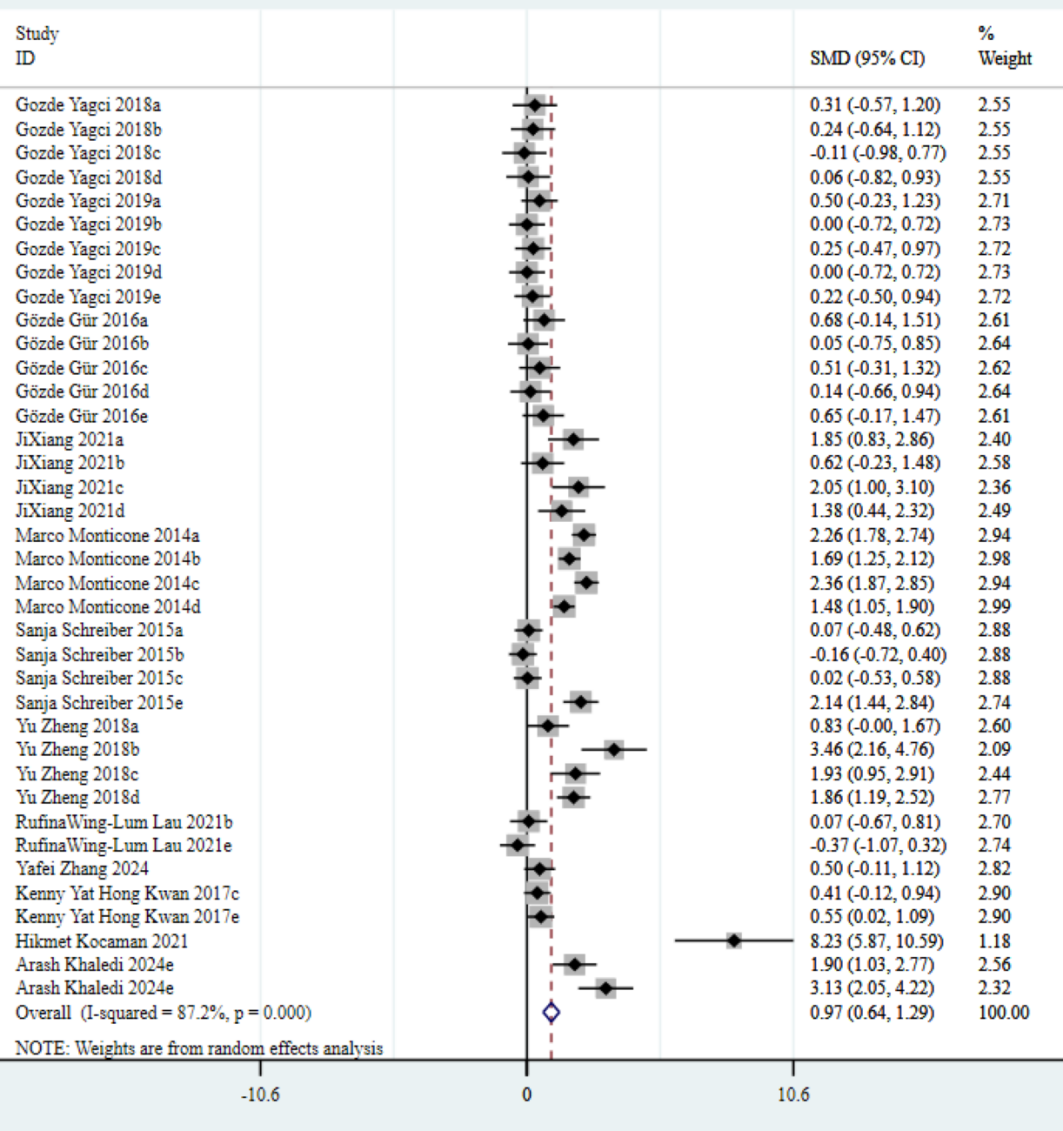
The forest plot of QoL.

Health-related quality of life was evaluated using the Scoliosis Research Society-22 (SRS-22) questionnaire. Exercise intervention was associated with a statistically significant improvement in quality of life among patients with AIS, yielding a moderate pooled effect size (*Hedge’s* = 0.73, 95% CI [0.38–1.09]). Substantial heterogeneity was observed across the included studies (I^2^ = 89.6%) (Fig. 4).

### Publication bias and sensitivity analysis

**Figure 5:**
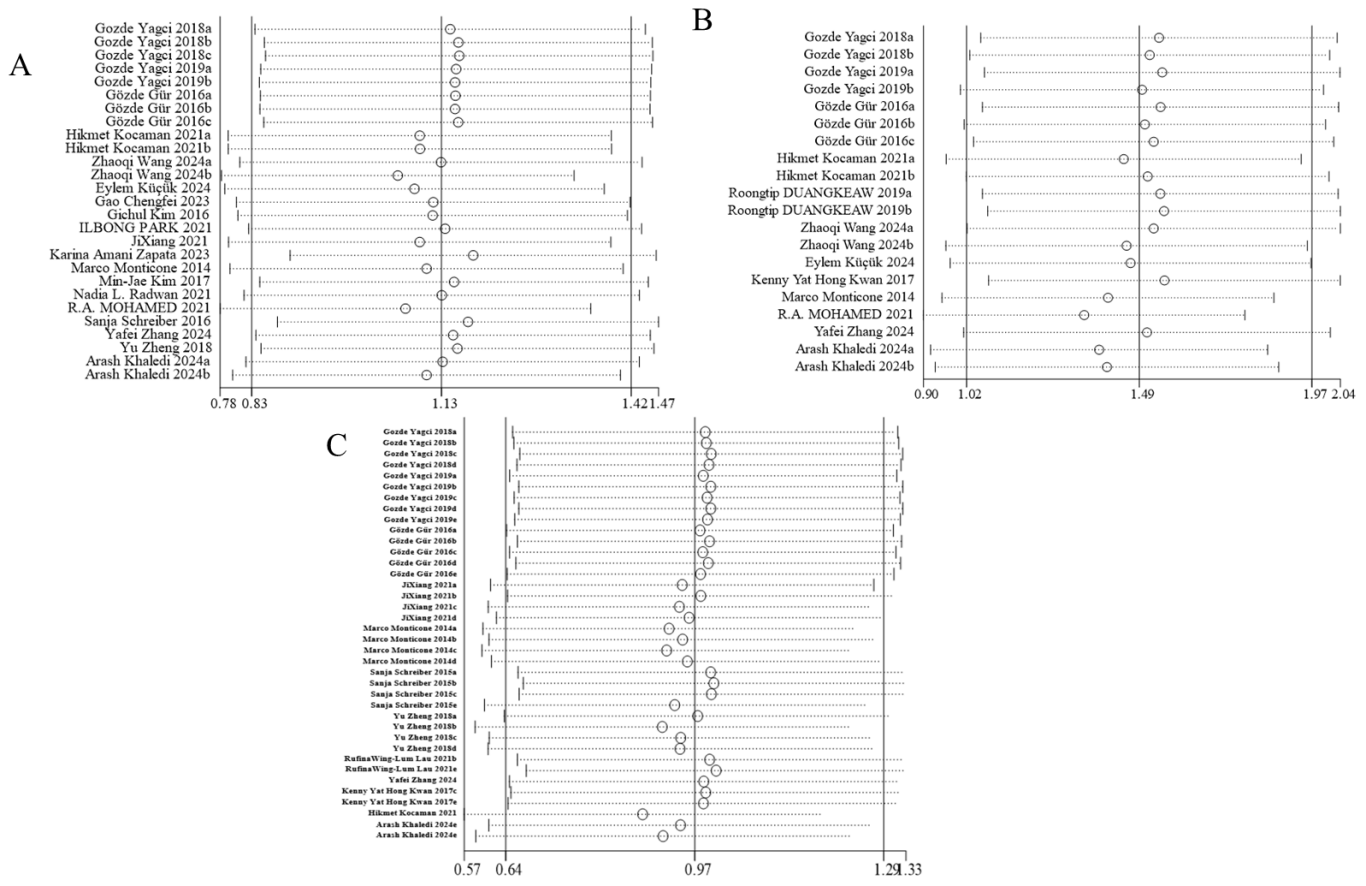
Sensitivity analysis plot. (A) Sensitivity analysis plot of cobb, (B) sensitivity analysis plot of ATR, (C) sensitivity analysis plot of QoL.

Sensitivity analyses revealed differential stability across outcome measures. Cobb angle demonstrated high robustness under varying model assumptions and parameter adjustments, with minimal fluctuation in effect estimates (Fig. 5). In contrast, trunk rotation angle (ATR) showed greater sensitivity to methodological variations such as measurement protocols or postural alignment, reflected by steeper slopes in sensitivity curves. Similarly, quality of life (QoL) outcomes were notably influenced by subjective assessment parameters, particularly under extreme scoring assumptions.

**Figure 6:**
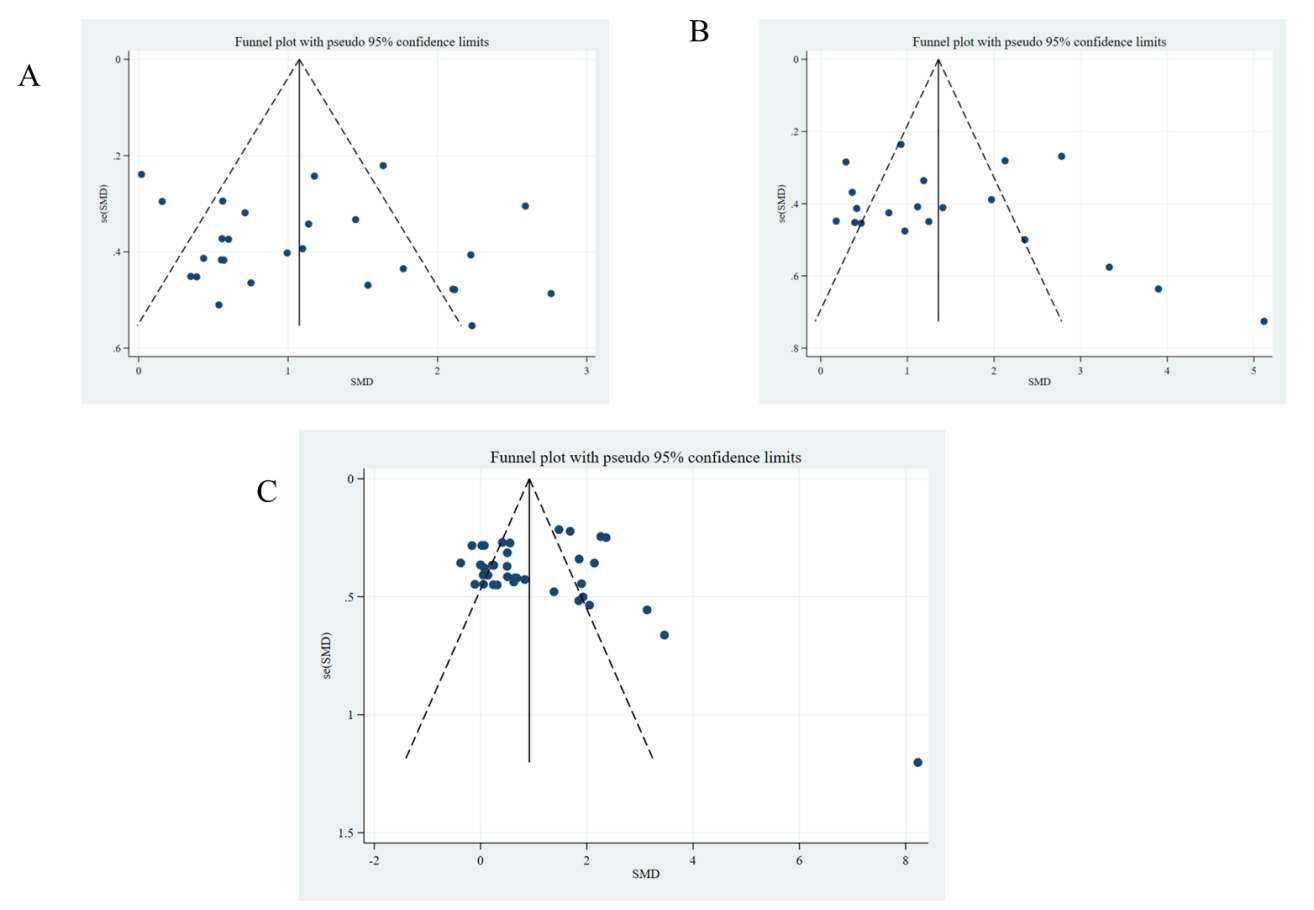
(A–C) Funnel plot.

Publication bias was assessed through funnel plots and Egger’s regression tests. Visual inspection of funnel plots indicated asymmetry suggestive of potential publication bias in Fig. 6. However, Egger’s test did not yield statistically significant intercepts for any outcome: cobb angle (*p* = 0.971 and 1.375), ATR (*p* = 1.60 and 0.87), or QoL (p1.26 and 0.62). These results suggest that while some asymmetry may exist, significant small-study effects were not consistently detected across analyses in Table 3.

### Effect of moderator variables

A random-effects meta-regression was conducted to evaluate the potential moderating effects of intervention characteristics, including training type, frequency, and total duration, on exercise treatment outcomes. None of these variables showed a statistically significant relationship with effect sizes (*p* > 0.05) in Table 4.

Subgroup analyses further revealed that interventions utilizing the Schroth method (*Hedge’s* = 1.304) and those combining exercise with orthotic management produced the greatest clinical improvements. Significant treatment effects were observed for both shorter-term (≤12 weeks) and longer-term (≥25 weeks) intervention durations in Table 5.

## Discussion

This systematic review and meta-analysis synthesized evidence from 24 studies to evaluate the effects of exercise-based interventions on Cobb angle, trunk rotation, and quality of life in patients with adolescent idiopathic scoliosis. The results indicate that exercise training significantly improves spinal curvature, reduces axial trunk rotation, and enhances quality of life in this population.

### Cobb

Subgroup analyses indicated that interventions incorporating Schroth therapy, particularly when combined with modalities such as orthotic bracing, kinesiology taping, or core stabilization training, yielded larger effect sizes. While these findings are consistent with previous meta-analytic evidence supporting the efficacy of Schroth therapy in reducing Cobb angles, they differ from studies reporting inconsistent outcomes for Schroth therapy combined with core stabilization training (Ceballos Laita et al., 2018; Dimitrijević et al., 2022).

The current synthesis highlights the therapeutic value of multimodal intervention approaches in adolescent idiopathic scoliosis. For instance, integrated protocols combining spinal mobilization with core stability training have been associated with statistically significant reductions in Cobb angle (Küçük, Öten & Coşkun, 2024). Similarly, improvements in thoracolumbar curvature, trunk rotation, and postural symmetry were observed following four months of combined orthotic bracing and core stabilization training (Yagci, Ayhan & Yakut, 2018).

Given that cosmetic improvement remains a primary treatment goal in scoliosis management, it is notable that both shorter (≤12 weeks) and longer (≥25 weeks) training durations significantly enhanced aesthetic outcomes. Moreover, a training frequency of five or more sessions per week was associated with improved physical tolerance and delayed Cobb angle progression (Wang, Lu & Wang, 2024).

### ATR

The ATR serves as a key clinical and prognostic marker for curve progression in AIS (Horne, Flannery & Usman, 2014). Evidence suggests that professionally supervised Schroth-based exercise programs can effectively reduce both Cobb angle and average trunk rotation in affected individuals (Kuru et al., 2016). Beyond modifying spinal deformity parameters, integrating Schroth therapy into routine care has been shown to improve back muscle endurance (Schreiber et al., 2015) and enhance trunk muscle strength following sustained intervention (Otman, Kose & Yakut, 2005).

**Table 3:** Egger’s test results.

**Indicators**	**Std_Eff**	**Coef**	**Std.Err**	**t**	***p*** > |t|	**[95% Conf.Interval]**	
Cobb	slope	0.5832786	0.5885376	0.99	0.331	−0.6288372	1.795394
	bias	1.419894	1.644921	0.86	0.396	−1.967884	4.807671
ATR	slope	0.504729	0.8540468	0.59	0.562	−1.289557	2.299015
	bias	2.336757	2.247806	1.04	0.312	−2.385707	7.059221
QoL	slope	0.6887057	0.5703221	1.21	0.235	−0.4679611	1.845372
	bias	0.6780902	1.613549	0.42	0.677	−2.594338	3.950519

**Table 4:** Results of meta-regression analysis.

**Moderator**	Cobb		ATR		QoL	
	t	*p*	t	*p*	t	*p*
Training type	−0.51	0.617	−0.91	0.378	−0.83	0.415
Duration	−1.22	0.234	1.95	0.069	0.23	0.820
Frequency	0.66	0.518	−0.39	0.698	1.03	0.308
Total sessions	2.38	0.026	1.08	0.298	0.80	0.427

**Table 5:** Results of subgroup analysis.

**Moderator**	**Sample size**			**SMD [95% CI]**			**I** ^**2**^ ** (%)**			***p***		
	**cobb**	**ATR**	**QoL**	**cobb**	**ATR**	**QoL**	**cobb**	**ATR**	**QoL**	**cobb**	**ATR**	**QoL**
Training type												
Schroth	8	5	8	1.304 [0.757, 1.851]	2.216 [1.001, 3.431]	1.056 [0.356, 1.755]	76.7	91.9	90.30	<0.001	<0.001	<0.001
Core training	4	2	12	0.777 [0.256, 1.298]	0.793 [0.308, 1.278]	1.165 [0.699, 1.63]	53.00	0.00	72.60	0.075	0.001	<0.001
Traditional rehabilitation	3	2	5	0.848 [0.109, 1.587]	1.441 [−0.118, 3.00]	0.001 [−0.331, 0.334]	74.10	91.8	0.00	0.009	0.07	0.851
Mixed intervention	8	7	9	1.252 [0.665, 1.840]	1.308 [0.671, 1.946]	1.186 [0.576, 1.795]	86.5	83.9	90.50	<0.001	<0.001	<0.001
Duration												
Short-term (≤12 weeks)	15	14	17	1.302 [0.92, 1.682]	1.307 [0.827, 1.787]	1.003 [0.537, 1.468]	72.5	82.30	81.50	<0.001	<0.001	<0.001
Mid-term (13–24 weeks)	1	2	5	0.579 [0.062, 1.097]	0.979 [0.365, 1.592]	0.259 [−0.026, 0.544]	0.00	53.30	0.00	0.028	0.002	0.842
Long term (≥25 weeks)	8	1	13	0.965 [0.446, 1.485]	3.851 [1.569, 6.134]	1.225 [0.665, 1.786]	84.30	89.0	91.7	<0.001	0.001	<0.001
Frequency												
Low frequency (1–2/week)	5	5	8	0.758 [0.192, 1.324]	0.847 [0.285, 1.41]	0.44 [−0.028, 0.909]	72.30	62.90	75.60	0.003	0.003	0.065
Medium frequency (3–4/week)	6	2	5	1.580 [1.116, 2.045]	4.041 [3.046, 5.036]	1.784 [1.129, 2.439]	52.4	46.6	63.9	0.026	<0.001	<0.001
High frequency (≥5/week)	13	10	22	1.062 [0.632, 1.492]	1.276 [0.743, 1.81]	0.981 [0.551, 1.41]	82.6	85.60	89.1	<0.001	<0.001	<0.001

Similarly, structured core stability training over 12 weeks has demonstrated efficacy in strengthening lumbar musculature, a finding supported by recent meta-analytic evidence highlighting the benefits of Pilates training on core muscle function (Ko & Kang, 2017). The interdependence between ATR and Cobb angle is well-established in the literature (Park, Jeon & Park, 2018), and the present analysis further supports this relationship (Ma et al., 2017), indicating that reductions in ATR correspond with decreases in Cobb angle.

Notably, improvements in trunk rotation were observed across intervention durations, including both shorter (<12 weeks) and longer (>25 weeks) protocols, aligning with existing evidence on effective treatment timelines.

### QoL

Numerous studies have established that exercise therapy effectively reduces the Cobb angle and meaningfully improves quality of life in adolescents with mild to moderate idiopathic scoliosis (Gür, Ayhan & Yakut, 2017; Ji, 2020; Monticone et al., 2014; Yagci, Ayhan & Yakut, 2018; Yagci & Yakut, 2019). QoL in this population is predominantly assessed using the Scoliosis Research Society-22 (SRS-22) questionnaire, a patient-reported outcome measure. Individuals with scoliosis commonly report higher back pain prevalence compared to healthy controls, largely attributable to asymmetric spinal loading, degenerative disc and facet joint changes, and muscular imbalances associated with disease progression.

While traditional exercise programs show limited efficacy in enhancing quality of life and alleviating pain, targeted approaches such as Schroth therapy, core stability training, and multimodal interventions incorporate specific exercises designed to improve postural control through neuromotor skill acquisition during functional tasks, while simultaneously optimizing deformity correction.

A methodological consideration pertains to the SRS-22 instrument. A notable ceiling effect has been reported for this scale, indicating potential limitations in its discriminative validity when assessing patients with varying degrees of severity, including those with mild curvature and preoperative cases Caronni, Zaina, Negrini (2014).

### Clinical implications

Based on the evidence synthesized, a stepped intervention framework can be recommended for adolescents with mild to moderate AIS. In the initial correction phase (≤12 weeks), supervised Schroth therapy delivered 1–2 times per week, integrated with core stabilization training, is effective for improving postural awareness and achieving early structural improvements. For individuals with curves exceeding 25° or those requiring further cosmetic and functional gains, a progressive control phase (≥25 weeks) is advised, involving Schroth therapy at a higher frequency (≥5 sessions/week), potentially combined with orthotic management to enhance and sustain corrective outcomes. Finally, transitioning to a tailored home-based exercise program supported by periodic clinical review and digital tools—such as wearable sensors for biofeedback and mobile applications for adherence monitoring—can help maintain long-term benefits and support patient engagement. This structured approach allows clinicians to adapt intervention intensity and duration according to disease severity and patient-specific goals.

### Heterogeneity within schroth-based interventions

The heterogeneity within our “Schroth” and “Mixed Intervention” subgroups warrants careful consideration. As Marchese, Ilhan & Pacey (2023) have documented, contemporary Schroth practitioners frequently integrate elements from multiple schools and combine Schroth with other therapeutic modalities. Our included studies exemplify this diversity: within the ‘Schroth’ category, protocols varied in intensity, frequency, and duration; within the ‘Mixed Intervention’ category, Schroth was combined with bracing, PNF, taping, laser acupuncture, HEP, or asymmetric stabilization exercises. This clinical heterogeneity likely contributed to the statistical heterogeneity observed in our pooled estimates and suggests that even refined categories like ‘standalone Schroth’ may encompass multiple distinct intervention variants. The differential effect sizes we observed across outcomes—with mixed interventions showing advantages for Cobb angle reduction, traditional rehabilitation for ATR improvement, and core training for QoL enhancement—further underscore that treatment effects are context-dependent and that ‘Schroth’ is not a one-size-fits-all solution. Future research should move toward even more precise intervention characterization, ideally comparing well-defined protocols (*e.g.*, traditional Schroth *vs.* PSSE Schroth *vs.* ScoliBalance) in head-to-head trials to determine whether theoretical differences translate into clinically meaningful outcome differences.

### Integration with recent evidence

The recent network meta-analysis provides complementary evidence to our findings. Their work, which included 22 studies and employed network meta-analysis to rank interventions, identified Schroth therapy as most effective for Cobb angle reduction, combined interventions for ATR improvement, and core stability training for QoL enhancement (Zhu et al, 2025). Our findings align with these rankings while extending them in important ways. Specifically, our analysis demonstrates that the effectiveness of these interventions is significantly moderated by how they are delivered—including session frequency, intervention duration, and whether they are combined with adjunctive therapies. For example, while Zhu et al. (2025) identified Schroth therapy as the top-ranked intervention for Cobb angle reduction, our subgroup analysis further specifies that optimal outcomes are achieved with either short-term (≤12 weeks, 1–2 sessions/week) or long-term (≥25 weeks, ≥5 sessions/week) protocols. This integration of comparative efficacy rankings with dose–response considerations provides a more complete evidence base for clinical decision-making.

## Limitation

The interpretation and generalizability of these findings are subject to several important limitations. (1) significant heterogeneity was observed across outcome measures, and the inclusion of only Chinese and English literature may have introduced language and selection bias, potentially omitting relevant evidence published in other languages. (2) Variability in intervention protocols—including mode, frequency, duration, and patient characteristics—contributed to considerable within-study and between-study inconsistency. (3) the available evidence is largely restricted to short-term outcomes (≤12 weeks or <6 months), with very few studies reporting follow-up data beyond one year; thus, the long-term efficacy of exercise interventions and their potential to modify the natural history of scoliosis remain uncertain. (4) methodological limitations were noted across several included studies, such as the use of non-randomized designs and inadequate reporting of blinding and allocation concealment, which may affect the internal validity of the findings. (5) the optimal weekly frequency, session duration, and exercise intensity for different intervention types were inconsistently reported across studies, limiting precise dose–response recommendations. (6) We acknowledge the publication of a recent network meta-analysis, which provides important comparative evidence on the relative efficacy of different exercise modalities for AIS (Zhu et al, 2025). Their work complements ours by offering a ranking of interventions based on indirect comparisons, while our review contributes a complementary perspective by systematically examining how intervention characteristics—including frequency, duration, and total sessions—moderate treatment effects across the same core outcomes. Together, these two approaches address different but equally important clinical questions: Zhu et al. (2025) help answer “which intervention is most effective?” while our analysis helps answer “how should that intervention be prescribed?” The existence of this prior work underscores the importance of situating our findings within the evolving evidence base and highlights the need for continued research that integrates both comparative efficacy and dose–response considerations. (7) Two eligible RCTs (Abbott, Möller & Gerdhem, 2013; Charalampidis et al., 2024) that met our inclusion criteria could not be incorporated into the quantitative synthesis due to the absence of extractable outcome data. Charalampidis et al. (2024) represents a methodologically rigorous trial comparing nighttime bracing with exercise interventions, and its exclusion may affect the comprehensiveness of our findings regarding comparative effectiveness. Abbott, Möller & Gerdhem (2013) provides the protocol for the ongoing CONTRAIS trial; future updates of this meta-analysis should incorporate these data when they become available to provide a more complete evidence synthesis.

## Conclusion and Future Research Recommendations

This meta-analysis synthesizes current evidence regarding the effects of exercise therapy on Cobb angle, ATR and QoL in adolescents with mild to moderate idiopathic scoliosis. The results indicate that exercise-based interventions significantly reduce Cobb angle, improve trunk rotation, and enhance health-related quality of life. The most pronounced benefits were observed with Schroth therapy, core stability training, and multimodal approaches combining orthotic management or neuromuscular techniques. By systematically analyzing how intervention characteristics—including frequency, duration, and total sessions—moderate treatment effects across different exercise modalities, this review provides a clinically actionable synthesis that complements recent network meta-analyses (*e.g.*, Zhu et al, 2025). While network meta-analyses offer valuable rankings of intervention efficacy, our work extends this knowledge by offering practical guidance on how to prescribe these interventions optimally. Together, these complementary approaches move the field beyond asking “if” or “which” exercise works toward addressing “how” to implement it effectively in clinical practice.

These findings support the SOSORT guideline recommendation that exercise therapy should serve as a foundational conservative treatment for patients with a Cobb angle below 45. Both short-term (≤12 weeks) and long-term (≥25 weeks) intervention durations demonstrated clinically meaningful effects. In practice, effective program design may involve Schroth therapy delivered as either a short-term (≤12 weeks, 1–2 sessions/week) or long-term (≥25 weeks, ≥5 sessions/week) regimen, ideally integrated with core muscle training or within a combined therapeutic framework.

To translate the current evidence into clinical practice, future research should prioritize several key directions. (1) establishing standardized dose-response protocols through high-quality randomized controlled trials is essential to define optimal frequency, intensity, time, and type parameters for different exercise modalities across varying curvature severities. (2) the development and validation of technology-enhanced rehabilitation platforms—such as AI-driven personalized training systems and remote monitoring tools with real-time biofeedback—are needed to improve exercise quality and long-term adherence. (3) multicenter, large-sample randomized controlled trials with extended follow-up periods exceeding one year should be conducted to compare the sustained efficacy of different intervention approaches, both alone and in combination with orthotic management, and to evaluate their ability to alter the natural progression of scoliosis. Addressing these priorities will provide the robust evidence necessary to refine clinical guidelines and optimize personalized rehabilitation strategies for AIS.

## Supplemental Information

10.7717/peerj.21355/supp-1 Supplemental Information 1 PRISMA checklist

10.7717/peerj.21355/supp-2 Supplemental Information 2 Search strategy

10.7717/peerj.21355/supp-3 Supplemental Information 3 Data

## Additional Information and Declarations

### Data Availability

The following information was supplied regarding data availability: The raw measurements are available in the Supplemental Files.
