# Supplementary material for: Effects of exercise interventions on adolescents with mild to moderate scoliosis: a meta-analysis
Source: PeerJ. 2026 Aug 6;14:e21355. doi: 10.7717/peerj.21355 (PMC13453138; doi:10.7717/peerj.21355)
Supplement: Supplementary file 2 [file peerj-14-21355-s002.docx]

Table S2. Search strategy.

| Pubmed | #1 "Adolescent"[Mesh]  #2 adolescent* OR teen* OR young people OR boy OR girl* OR teenager* OR child OR young adult[Title/Abstract]  #3 #1 OR #2  #4 "Scoliosis"[Mesh]  #5 idiopathic scoliosis OR adolescent idiopathic scoliosis OR AIS[Title/Abstract]  #6 #4 OR #5  #7 "Exercise"[Mesh]  #8 train* OR sport* OR physical activity OR motion OR movement OR locomotion OR athletic* OR gymnastic* OR motor OR physiotherapeutic scoliosis specific exercis OR PSSE OR Schroth OR Barcelona Scoliosis Physical Therapy School OR BSPTS OR Lyon OR scientific exercises approach scoliosis OR SEAS OR DoboMed OR Side-shift OR functional individual therapy scoliosis OR FITS OR physiotherapy[Title/Abstract]  #9 #7 OR #8  #10 #3 AND #6 AND #9 |
| --- | --- |
| Web of Science | #1 TS=Adolescent  #2 adolescent*[TIAB] OR teen*[TIAB] OR young people[TIAB] OR boy[TIAB] OR girl*[TIAB] OR teenager*[TIAB] OR child[TIAB] OR young adult[TIAB]  #3 #1 AND #2  #4 TS=Scoliosis  #5 idiopathic scoliosis OR adolescent idiopathic scoliosis OR AIS[TIAB]  #6 #4 AND #5  #7 TS=Exercise  #8 train* OR sport* OR physical activity OR motion OR movement OR locomotion OR athletic* OR gymnastic* OR motor OR physiotherapeutic scoliosis specific exercis OR PSSE OR Schroth OR Barcelona Scoliosis Physical Therapy School OR BSPTS OR Lyon OR scientific exercises approach to scoliosis OR SEAS OR DoboMed OR Side-shift OR functional individual therapy of scoliosis OR FITS OR physiotherapy[TIAB]  #9 #7 OR #8  #10 #3 AND #6 AND #9 |
| EBSCO | ( adolescent OR youth OR teenager OR teen OR child OR children ) AND ( Scoliosis OR scoliosis ) AND ( Exercise OR train OR sport OR physical activity OR motion OR movement OR locomotion ) AND randomized controlled trials |
